# The effect of forskolin and the role of Epac2A during activation, activity, and deactivation of beta cell networks

**DOI:** 10.3389/fendo.2023.1225486

**Published:** 2023-08-28

**Authors:** Maša Skelin Klemen, Jurij Dolenšek, Lidija Križančić Bombek, Viljem Pohorec, Marko Gosak, Marjan Slak Rupnik, Andraž Stožer

**Affiliations:** ^1^ Institute of Physiology, Faculty of Medicine, University of Maribor, Maribor, Slovenia; ^2^ Faculty of Natural Sciences and Mathematics, University of Maribor, Maribor, Slovenia; ^3^ Alma Mater Europaea, European Center Maribor, Maribor, Slovenia; ^4^ Center for Physiology and Pharmacology, Medical University of Vienna, Vienna, Austria

**Keywords:** pancreas, tissue slices, beta cells, calcium imaging, amplifying pathway, forskolin, Epac2A KO, intercellular network

## Abstract

Beta cells couple stimulation by glucose with insulin secretion and impairments in this coupling play a central role in diabetes mellitus. Cyclic adenosine monophosphate (cAMP) amplifies stimulus-secretion coupling via protein kinase A and guanine nucleotide exchange protein 2 (Epac2A). With the present research, we aimed to clarify the influence of cAMP-elevating diterpene forskolin on cytoplasmic calcium dynamics and intercellular network activity, which are two of the crucial elements of normal beta cell stimulus-secretion coupling, and the role of Epac2A under normal and stimulated conditions. To this end, we performed functional multicellular calcium imaging of beta cells in mouse pancreas tissue slices after stimulation with glucose and forskolin in wild-type and Epac2A knock-out mice. Forskolin evoked calcium signals in otherwise substimulatory glucose and beta cells from Epac2A knock-out mice displayed a faster activation. During the plateau phase, beta cells from Epac2A knock-out mice displayed a slightly higher active time in response to glucose compared with wild-type littermates, and stimulation with forskolin increased the active time via an increase in oscillation frequency and a decrease in oscillation duration in both Epac2A knock-out and wild-type mice. Functional network properties during stimulation with glucose did not differ in Epac2A knock-out mice, but the presence of Epac2A was crucial for the protective effect of stimulation with forskolin in preventing a decline in beta cell functional connectivity with time. Finally, stimulation with forskolin prolonged beta cell activity during deactivation, especially in Epac2A knock-out mice.

## Introduction

Insulin serves as an important regulator of nutrient homeostasis and a lack of its effects, either due to beta cell failure or resistance in peripheral tissues, or both, results in diabetes mellitus, which affects more than 537 million people worldwide (1). 90% of all diabetes cases are type 2 diabetes mellitus (T2DM), which is characterized by impaired insulin secretion, insulin resistance, and obesity (2).

Pancreatic beta cells act as sensors for blood glucose changes and respond with altering insulin secretion via a process termed stimulus-secretion coupling, by activation of the triggering and amplifying pathways (3, 4). The triggering pathway is initiated by glucose entry into beta cells through glucose transporters (GLUT), followed by glucose metabolism and intracellular ATP concentration increase, which results in decreased open probability of the K_ATP_ channels. The subsequent decrease in potassium efflux depolarizes the cell membrane, leading to an increase in permeability of VDCC allowing Ca^2+^ ions influx into the cytoplasm (5). Being the central intracellular messenger, Ca^2+^ activates exocytosis of insulin-containing granules (3, 6, 7).

The triggering pathway is augmented and fine-tuned by amplifying pathways which start with either (i) an increase in [Ca^2+^]_IC_ (8), (ii) GPCRs activation (9), or (iii) phospholipase C (PLC) activation (10). The result of either [Ca^2+^]_IC_ elevation or GPCR activation is enhanced adenylyl cyclase activity, which increases the concentration of cAMP. Consequently, cAMP enhances fusion of insulin-containing vesicles with plasma membrane via both a protein kinase A (PKA)-dependent pathway and activation of the guanine nucleotide exchange protein 2 (Epac2A) (11–13), whose role is not fully elucidated yet.

When blood glucose levels are high, PKA-dependent amplification mostly increases the second phase of insulin secretion (14) by inhibiting K_ATP_ channel activity in an ADP-dependent manner (15), as well as inhibits K^+^ currents through voltage dependent K_V_1.4 channels (16, 17). Further enhancement of Ca^2+^ signaling can be mediated through VDCCs (18–20) or Ca^2+^ mobilization from internal stores through both IP3 and ryanodine receptors (18–22). In addition, PKA increases the number of insulin-containing granules that are highly sensitive to Ca^2+^ (23, 24), the mobility and replenishment of the readily releasable pool (RRP) of insulin-containing granules (11), and the overall sensitivity of the secretory machinery to [Ca^2+^]_IC_ (25).

Along with influences on PKA, cAMP affects insulin secretion via Epac2A (13, 26–28), which has been implicated in both the first and the second phase of insulin secretion (13, 14). While the Epac1 isoform is ubiquitous, Epac2A is located mainly in the neural, endocrine, and neuroendocrine tissue (27, 29), and seemingly predominant in mouse islets (30). The effects of Epac2A on insulin secretion are mediated in a myriad of ways. It has been proposed that GLP-1-induced mitochondrial ATP synthesis, which contributes to closure of K_ATP_ channels, is partially mediated by Epac2A (31). Furthermore, Epac2A contributes to increased activity of the enzyme glucokinase, increasing the production of mitochondrial metabolism substrates (32). Under the influence of Epac2A, K_ATP_ channels exhibit increased ATP sensitivity, facilitating membrane depolarization (33, 34). Epac2A also influences [Ca^2+^]_IC_ dynamics by RYR2 sensitization, thus encouraging Ca^2+^ release from the endoplasmic reticulum Ca^2+^ stores upon stimulation with increased [Ca^2+^]_IC_ via the Rap protein and PLC-ϵ (35), a process designated as calcium-induced calcium release (36, 37). Epac2A may also augment Ca^2+^ influx by influencing VDCCs via Rim2α (38, 39). Downstream from [Ca^2+^]_IC_, Epac2A increases the size of the readily releasable pool of granules through Epac2/Rap1 signaling. It also influences docking and priming of granules when complexed with the Rab3-interacting molecule Rim2α (39), and stimulates insulin granule acidification, an important step in granule priming by regulating Cl^-^ influx (12). Previous research on mice lacking Epac2A (Epac2A KO) has demonstrated a propensity for obesity (40), a diminished first phase of insulin secretion (13), and impaired glucose-stimulated insulin secretion under diet-induced insulin resistance (41).

Besides exhibiting complex and intertwined intracellular signaling pathways, beta cells are intrinsically highly heterogeneous and operate in a multicellular environment. They communicate with each other through different mechanisms, whereby the electrical coupling through gap-junctions is recognized as the main synchronizing agent that facilitates the spreading of depolarization and Ca^2+^ waves across the islets (42–48). This enables beta cells to operate in a coordinated manner, which was found essential for the normal control of hormone secretion (3, 49–53). However, due to their multifaced cell-to-cell variability, the resulting multicellular activity is not completely synchronized and manifests itself in the form of non-stationary waves, guided by the intrinsic islet heterogeneity (54–59). In recent years, graph-theoretical approaches have been used as a powerful methodology to quantify this non-trivial spatiotemporal behavior within the islets (48, 60–63). By this means, functional connectivity networks are typically constructed based on statistical similarity of Ca^2+^ traces and previous research has shown that the beta cells form architectures with high communication capacities that are locally segregated into functional subcompartments (63–65). Moreover, beta cell networks are highly heterogeneous and some very well-connected cells, which were identified as metabolically highly active, are believed to be crucial for routing information between cells (66–69). However, the role of different signaling pathways in the coordination of the collective cellular rhythms in islets, and particularly the role of cAMP signaling are incompletely understood. Previous studies suggest that intracellular cAMP concentration influences gap-junctional coupling between beta cells (70–74). Recently, PKA and Epac2A were reported to increase Cx36 gap-junctional coupling by different means (51, 75). Noteworthy, cAMP serves as a strong modulator of intercellular communication and the effect can be controlled by either arm of the incretin signaling cascade (76–79). Therefore, it remains of great interest to explore in more detail how neurohormonal amplifying pathways affect multicellular beta cell activity.

In this study, we simultaneously recorded [Ca^2+^]_IC_ oscillations in a large number of beta cells in acute pancreas tissue slices with single-cell resolution to assess the role of Epac2A knockout during activation, activity, and deactivation, as well as the effects on intercellular coupling assessed by functional connectivity analyses. We further analyzed the effects of the cAMP-elevating diterpene forskolin on [Ca^2+^]_IC_ and determine the contribution of Epac2A in mediating these effects. To this end, we performed experiments using forskolin in otherwise substimulatory as well as in stimulatory glucose conditions, in both Epac2A KO mice and their WT littermates.

## Materials and methods

### Ethics statement

The study was conducted as per the protocol approved by the Administration for Food Safety, Veterinary Sector and Plant Protection of the Republic of Slovenia (permit numbers: U3440-1-61/2009/2 and U34401-12/2015/3) and complied with all national and European recommendations relating to care and work with experimental animals to minimize animal discomfort.

### Animals, tissue slice preparation and dye loading

Epac2A KO mice (Rapgef4^tm1.1Sse^) were generated by Professor Susumu Seino of Kobe University as described previously (13). In short, a loxP site was inserted into exon 1 and a floxed neo cassette was inserted downstream of exon 1. Cre-mediated recombination was used to delete a portion of exon 1. The absence of protein product in pancreatic islet cells was confirmed by RT-PCR analysis. Epac2A HET mice with C57BL/6 background were maintained to obtain Epac2A WT and Epac2A KO littermates. The study was conducted on 5 Epac2A WT and 5 Epac2A KO mice.

Acute pancreas tissue slices were obtained from 12-24 weeks old Epac2A KO mice and their WT littermates of either sex were kept in individually ventilated cages (Allentown LLC, USA) with a 12:12 hours light: dark schedule, as described previously (43, 80). In brief, mice were sacrificed using high concentration of CO_2_, and the abdominal cavity was accessed via laparotomy. The pancreas was injected with low-melting point 1.9% agarose (Lonza, USA) kept at 40°C dissolved in extracellular solution (ECS, consisting of (in mM) 125 NaCl, 26 NaHCO_3_, 6 glucose, 6 lactic acid, 3 myo-inositol, 2.5 KCl, 2 Na-pyruvate, 2 CaCl_2_, 1.25 NaH_2_PO_4_, 1 MgCl_2_, 0.5 ascorbic acid) with access through the proximal common bile duct, which was clamped distally at the major duodenal papilla. Following injection, the pancreas was cooled with ice-cold ECS and extracted from the abdominal cavity. A vibratome (VT 1000 S, Leica) was used to create 140 µm thick tissue slices, which were collected at room temperature in HEPES-buffered saline (HBS, consisting of (in mM) 150 NaCl, 10 HEPES, 6 glucose, 5 KCl, 2 CaCl_2_, 1 MgCl_2_; titrated to pH=7.4 using 1 M NaOH). The slices were incubated in the dye-loading solution (6 µM Oregon Green 488 BAPTA-1 AM (OGB-1, Invitrogen), 0.03% Pluronic F-127 (w/v), and 0.12% dimethylsulphoxide (v/v) dissolved in HBS) for 50 minutes at RT. All chemicals were obtained from Sigma-Aldrich (St. Louis, Missouri, USA) unless otherwise specified.

### Stimulation protocol and calcium imaging

Individual tissue slices were exposed to single square pulse-like stimulation per islet in a perifusion system containing carbogenated ECS at 37°C. Regardless of the number of islets observed on an individual tissue slice, each slice was stimulated with only a single stimulatory protocol. In control experiments, slices were exposed to 12 mM glucose for 25 minutes, followed by incubation in a solution with substimulatory 6 mM glucose concentration until apparent cessation of activity. In experiments using forskolin slices were exposed to 6 mM or 12 mM glucose for 5 and 15 min, respectively, followed by the addition of 10 µM forskolin for 10 min and the subsequent substimulatory 6 mM glucose concentration again until the deactivation of cells. Calcium imaging was performed on a Leica TCS SP5 DMI6000 CS inverted confocal system (20X HC PL APO water/oil immersion objective, NA 0.7) and a Leica TCS SP5 AOBS Tandem II upright confocal system (20x HCX APO L water immersion objective, NA 1.0). Acquisition frequency was set to 1-2 Hz at 512 x 512 pixels. For a more precise quantification of [Ca^2+^]_IC_ oscillations in the phases of sustained activity, a resolution of 10 Hz at 256 x 256 pixels was maintained throughout the whole stimulation protocol. The laser power was adapted to avoid photobleaching and prolong the maximum time of recording, while maintaining a satisfactory signal strength. Cells were imaged at the approximate depth of 15 µm or more to avoid recording from cells at the surface. The optical section thickness was kept at about 4 µm to assure recordings from a single cell only. OGB-1 was excited by an argon 488 nm laser line and emitted fluorescence was detected by Leica HyD hybrid detector in the range of 500-700 nm (all from Leica Microsystems, Germany), as described previously (43, 46, 81).

### Processing of Ca^2+^ signals and beta cell functional network analysis

Individual ROIs were selected manually with respect to cell morphology using a high-resolution image, maximal projection, or frame average. Time series were exported using custom-made software (ImageFiltering, copyright Denis Špelič). Custom-made Matlab and Phyton scripts were used for subsequent off-line analysis. Fluorescence signals were expressed as F/F_0_, the ratio of the fluorescence signal (F) at a certain time point of the experiment relative to the initial fluorescence (F_0_). A combination of exponential and linear fit was applied to correct the time series data for photobleaching. Time series with distorted signals due to movement artefacts were removed from further analysis. Also excluded were all series with evident non-beta cell-like patterns, such as low-glucose-active and high-glucose-inactive cells, or constantly active or sporadically active with various oscillation frequencies, suggestive of other islet cell types (82–86). Beginnings of [Ca^2+^]_IC_ increases and decreases after exposure to high and low glucose were used to manually select activation and deactivation times, respectively.

A zero-lag digital filter was used on Ca^2+^ traces to extract the classical signaling parameters in the phase of sustained activity, with the purpose of removing noise and possible low frequency baseline variations. Typical cutoff frequencies were determined empirically in ranges of 0.02-0.04 Hz and 1-2 Hz for the lower and upper boundary, respectively, as described previously (62, 87). This was followed by time series binarization so that the values from the onset to the end of individual oscillations were 1, and values between the oscillations were 0. Specifically, this process involved detecting the onsets, peaks, and endings of oscillations in a given time series. The onsets were determined by analyzing the derivative (slopes) of the time series, identifying significant changes, i.e., beginning of Ca^2+^ increases. The peaks represent the maximum signal intensity within each oscillation. The endings were defined as the points where the signal intensity decays to 50% of the maximum value in a given oscillation. These steps are repeated throughout the whole time series, i.e., for all oscillations. Binarized time series data was then used to characterize the oscillatory activity based on the following parameters: (i) average frequencies, (ii) average durations of oscillations, and (iii) relative active times of individual cells. The latter represents the average fraction of time that cells spend in an active state with increased Ca^2+^ and serves as a suitable measure of the cell’s overall activity (81). The variability of the activation delays was measured by calculating delays relative to the first responding cells in an islet (any-cell-first-responder delays) or to account for the inter-islet differences, dividing the data with the respective islet median delay (relative any-cell-first-responder delays).

To assess the collective beta cell activity in each islet, we generated functional connectivity networks. By this means, nodes represent individual beta cells, and their locations correspond to physical positions of cells in tissue slices. Connections between node pairs were created based on the temporal similarity of the measured Ca^2+^ dynamics, as evaluated through the calculation of the correlation coefficient, as explained previously (65). We have used variable thresholds to extract the connectivity matrix, so that the average node degree in each islet, i.e., the average number of connections per cell <*k*>, in the first part in the sustained activity phase (12 mM glucose only) was <*k*>=8 and the same connectivity threshold was then used in the second part in the phase of sustained activity (either 12 mM glucose or 12 mM glucose + 10 µM forskolin) in the same islet. In this way a comparison between different islets was facilitated as well as a direct evaluation of the changes between both intervals. The extracted functional networks were analyzed with common network metrics. Specifically, the average correlation coefficient along with the average node degree were used to evaluate the average level of beta cell synchronicity in the given islet. Modularity was used to characterize the level of functional segregation, i.e., the extent of partitioning into smaller subpopulations. It measures how well the nodes in a network can be clustered into communities and is computed as the fraction of the edges that exist within communities minus the expected fraction of edges that would exist within communities if the network were random. The values of modularity span between 0 and 1, where 0 means that the nodes in the network are not clustered at all, while a value of 1 means that the nodes are perfectly divided into functional submodules. Moreover, we computed the relative largest component to quantify the level of the network’s functional integration. The largest component of a network is the connected subgraph with the most nodes and is computed by starting with a single node and then iteratively adding all of the nodes that are connected to it. This process is repeated until no more nodes can be added. If the largest component is very small (i.e., close to zero), then the network is likely to be fragmented and difficult to traverse, whilst high values (i.e., close to 1) indicate a very cohesive and integrated architecture. For details, see (63, 65).

## Results

**Figure 1:**
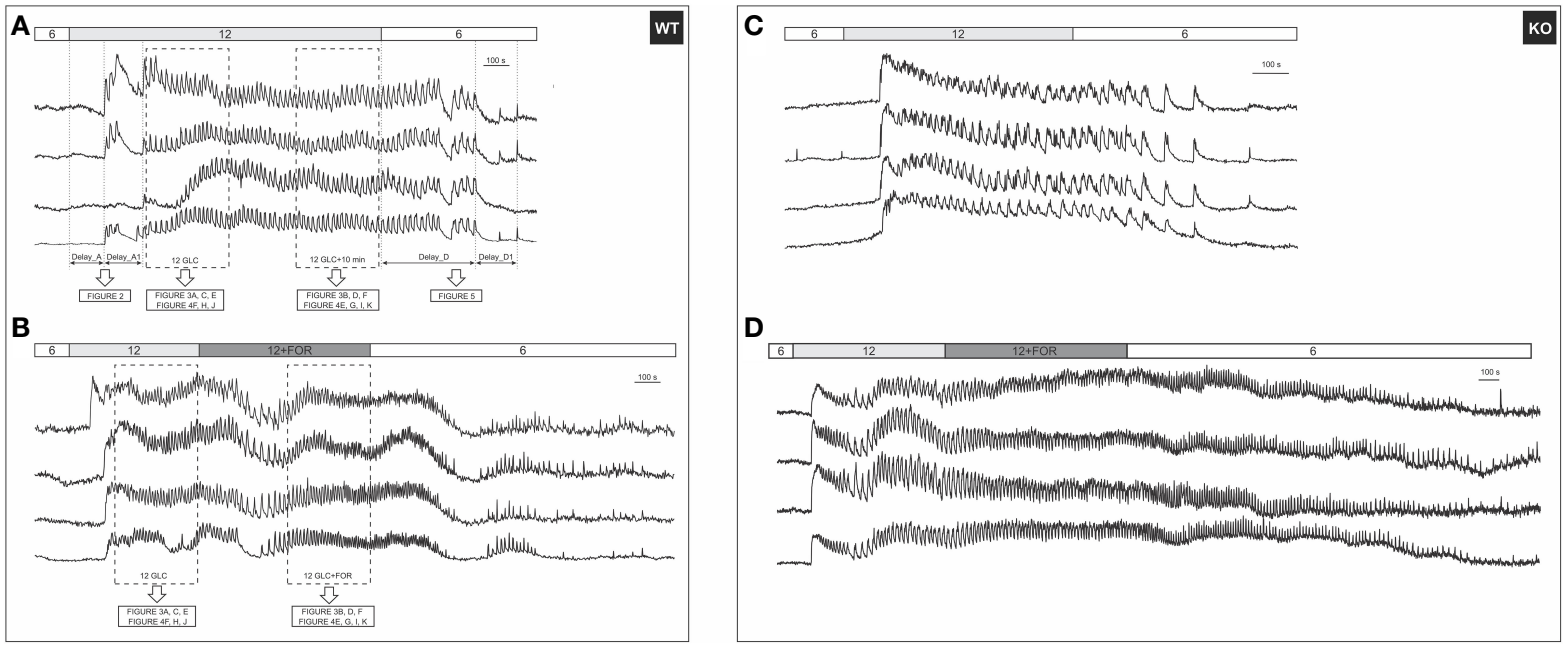
A typical beta cell response to glucose and forskolin. **(A)** 12 mM glucose induced a transient increase in [Ca^2+^]_IC_, followed by [Ca^2+^]_IC_ oscillations during the plateau phase that ceased after stimulus withdrawal. Responses of 4 cells from the same WT islet are shown. Activation (Delay_A) and deactivation (Delay_D) delays were defined as indicated in the figure; **(B)** [Ca^2+^]_IC_ activity after addition of 10 µM forskolin in 4 cells from the same WT islet. Rectangles indicate intervals used in subsequent analysis of the plateau phase, the results of which are shown in indicated figures. Note that the prolonged stimulation with glucose only **(A)** served as a control for possible time effects, since forskolin was added to glucose after a stable plateau was achieved in response to glucose **(B)**; **(C)** Responses to 12 mM glucose of 4 cells from the same KO islet are shown; **(D)** [Ca^2+^]_IC_ activity after addition of 10 µM forskolin in 4 cells from the same KO islet. The analysis methodology was the same as demonstrated in **(A, B)**.

A typical beta cell response to glucose stimulation features three subsequent phases of [Ca^2+^]_IC_ activity, i.e., the activation, the plateau, and the deactivation phase (**Figure 1** (43, 81)). To enhance the reader’s comprehension of the recorded signals, an additional time-lapse video was included (**Video S1**). Below, we present the results according to this order of the three response phases.

### The effect of forskolin and the role of Epac2A in activation of beta cells

**Figure 2:**
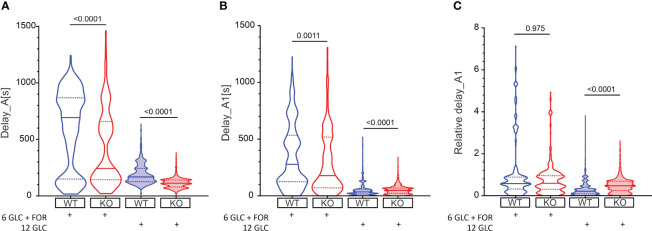
The effect of forskolin and the role of Epac2A during activation of beta cells from WT and KO mice. **(A)** The activation delays (Delay_A) after stimulation with either 6 mM glucose + 10 µM forskolin or 12 mM glucose in WT and Epac2A KO mice. Note that the 6 mM glucose failed to elicit a response in beta cells. 1^st^ quartile/median/3^rd^ quartile (Q1/M/Q3, in seconds): 151/693/868 (6 mM glucose + 10 µM forskolin in WTs), 144/243/659 (6 mM glucose + 10 µM forskolin in KOs), 127/168/243 (12 mM glucose in WTs), and 81/112/142 (12 mM glucose in KOs); **(B)** The any-cell-first-responder delays after stimulation with either 6 mM glucose + 10 µm forskolin or 12 mM glucose in WT and Epac2A KO mice. Q1/M/Q3 (in seconds): 126/278/533 (6 mM glucose + 10 µM forskolin in WTs), 70/178/518 (6 mM glucose + 10 µM forskolin in KOs), 17/35/58 (12 mM glucose in WTs), and 22/48/69 (12 mM glucose in KOs); **(C)** Relative any-cell-first-responder delays after stimulation with either 6 mM glucose + 10 µm forskolin or 12 mM glucose. Q1/M/Q3: 0.32/0.58/0.90 (6 mM glucose + 10 µM forskolin in WTs), 0.31/0.59/0.95 (6 mM glucose + 10 µM forskolin in KOs), 0.009/0.21/0.40 (12 mM glucose in WTs), and 0.24/0.47/0.69 (12 mM glucose in KO). Data pooled from the following number of cells/islets: 388/6 (6 mM glucose + 10 µM forskolin in WTs), 428/8 (6 mM glucose + 10 µM forskolin in KOs), 1373/24 (12 mM glucose in WTs), 1375/22 (12 mM glucose in KOs). Data were analyzed using Mann-Whitney U test, p values are indicated on graphs.

Beta cells were inactive in 6 mM glucose in both WT and Epac2A KO mice (**Figure 1**). We tested whether stimulation by forskolin produced a shift in glucose sensitivity, and whether this effect was Epac2A-dependent (**Figures 2**, **S1**). 10 µM forskolin added to 6 mM glucose activated beta cells in a qualitatively similar manner as 12 mM glucose. In quantitative terms, the delays between the stimulus onset and the activation of cells after the forskolin stimulus were smaller in KO mice (243 s vs. 693 s, delay_A). Surprisingly, the absence of Epac2A affected the activation properties in a similar manner even during stimulation with glucose only, as the activation delays were shorter in Epac2A KO mice (112 s vs. 168 s, **Figure 2**), suggesting a possible inhibitory role of Epac2A during the activation phase at both basal conditions and during forskolin-stimulated conditions.

We have shown previously that the glucose activation of beta cells was relatively heterogeneous (**Figure 1**), and that the differences in activation delays were strongly glucose-dependent (81). In this vein, we assessed heterogeneity during activation by calculating delays of every cell relative to the first-responding cell in an islet (i.e., any-cell-first-responder delays, delay_A1, **Figure 2B**). The lack of Epac2A enlarged the any-cell-first-responder delay after stimulation with 12 mM glucose by 26% (48 s in KO vs. 35 s in WT mice), in contrast to a decrease by 36% after stimulation with forskolin plus 6 mM glucose (178 s in KO vs. 278 s in WT mice). Importantly, to account for the inter-islet differences and the shorter activation in Epac2A KO mice, the data were normalized with the respective islet median delays (**Figure 2C**), the Epac2A deletion increased activation heterogeneity after stimulation with 12 mM glucose (0.47 in KO and 0.21 in WT mice) and did not have a significant effect after stimulation with forskolin plus 6 mM glucose. In sum, Epac2A seems to contribute to a less heterogeneous activation during stimulation with glucose.

### The effect of forskolin and the role of Epac2A during the plateau phase

We further explored the effect of stimulation by forskolin and the specific role of Epac2A during the plateau phase by analyzing the classical and network functional parameters.

#### Classical functional parameters

**Figure 3:**
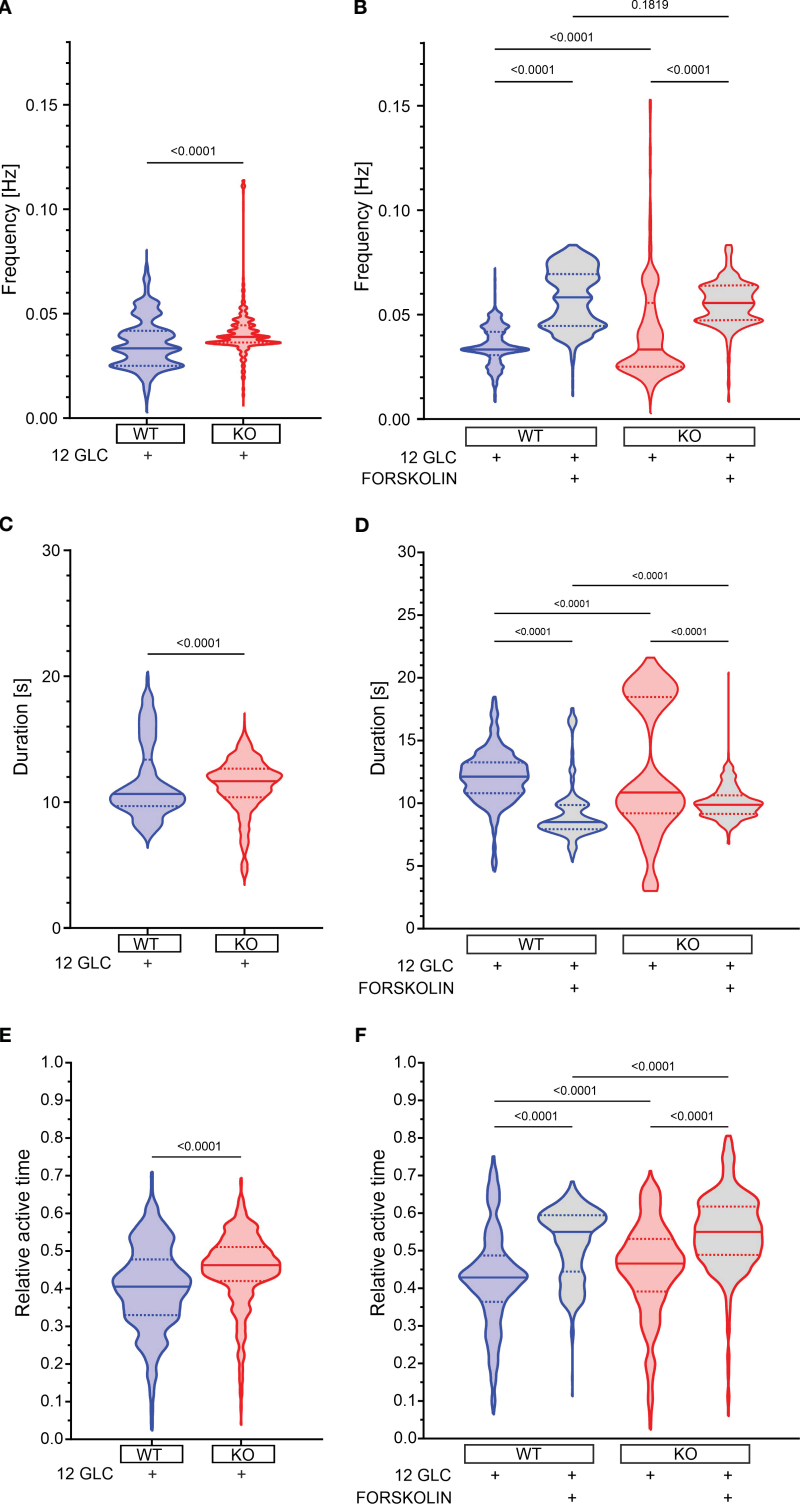
The effect of forskolin and the role of Epac2A during the plateau phase of response to glucose. **(A)** The oscillation frequency after stimulation with 12 mM glucose. 1^st^ quartile/median/3^rd^ quartile (Q1/M/Q3) in Hz: 0.025/0.033/0.042 (in WTs) and 0.036/0.039/0.044 (in KOs); **(B)** The oscillation frequency after either prolonged stimulation with 12 mM or addition of 10 µm forskolin. Q1/M/Q3 in Hz: 0.031/0.033/0.042 (12 mM glucose in WTs), 0.045/0.058/0.069 (12 mM glucose + 10 µM forskolin in WTs), 0.025/0.033/0.056 (12 mM glucose in KOs) and 0.047/0.056/0.064 (12 mM glucose + 10 µM forskolin in KOs); **(C)** The oscillation duration after stimulation with 12 mM glucose. Q1/M/Q3 in seconds: 9.7/10.7/13.4 (in WTs) and 10.4/11.7/12.7 (in KOs); **(D)** The oscillation duration after either prolonged stimulation with 12 mM or addition of 10 µm forskolin. Q1/M/Q3 in seconds: 10.8/12.1/13.3 (12 mM glucose in WTs), 7.9/8.5/9.9 (12 mM glucose + 10 µM forskolin in WTs), 9.2/10.9/18.5 (12 mM glucose in KOs), and 9.2/9.9/10.6 (12 mM glucose + 10 µM forskolin in KOs); **(E)** The relative active time after stimulation with 12 mM glucose. Q1/M/Q3: 0.33/0.41/0.48 (in WTs) and 0.42/0.46/0.51 (in KOs); **(F)** The relative active time after either prolonged stimulation with 12 mM or addition of 10 µm forskolin. Q1/M/Q3: 0.36/0.43/0.49 (12 mM glucose in WTs), 0.44/0.55/0.59 (12 mM glucose + 10 µM forskolin in WTs), 0.39/0.47/0.53 (12 mM glucose in KOs) and 0.49/0.55/0.62 (12 mM glucose + 10 µM forskolin in KOs). Data pooled from the following number of cells/islets: 687/11 (12 mM glucose in WTs), 656/11 (12 mM glucose + 10 µM forskolin in WTs), 548/7 (12 mM glucose in KOs), and 631/9 (12 mM glucose + 10 µM forskolin in KOs). Data were analyzed using Mann-Whitney U test (for 2 samples) or one-way ANOVA on ranks (Kruskal-Wallis test) followed by Dunn’s multiple comparisons test for more than 2 samples), p values are indicated on graphs.

12 mM glucose evoked repetitive fast [Ca^2+^]_IC_ oscillations (**Figure 1**). Relative active time of a cell (defined as the percentage of oscillatory activity that the cells spent at a [Ca^2+^]_IC_ > 50% of the oscillation amplitude, was 41% at a frequency of 0.033 Hz and an oscillation duration of 10.7 s (**Figures 3A, C, E**). Epac2A deletion raised the active time by 12% (AT = 46%), by increasing both oscillation duration (11.7 s) and frequency (0.039 Hz).

Next, we tested the effect of forskolin. In WT mice, 10 µM forskolin added to the stimulatory 12 mM glucose raised the active time further by 28% (from 43% to 55%, **Figures 3F**, **S3C**). There was a considerable drop in oscillation duration by 30% (8.5 s vs. 12.1 s, **Figures 3D**, **S3B**), but active time nevertheless rose due to a large increase in frequency by 75% (0.058 Hz vs. 0.033 Hz, **Figures 3B**, **S3A**), corroborating the importance of analyzing the active time rather than the frequency or the durations only (88). In Epac2A KO mice the frequency also increased, oscillation duration decreased, and the active time increased to a similar extent and a similar absolute value as in WT mice (increase by 17%, i.e. from 47% to 55% **Figures 3F**, **S3**). These changes in frequency, duration and AT were not due to prolonged exposure to the stimulating glucose concentration alone, as significantly smaller changes were observed between the two time intervals analyzed in cells stimulated with high glucose only (**Figures S2**, **S3**). To provide a more detailed insight into how individual islets responded to glucose only and forskolin, we additionally present in **Figure S3** how active time, duration, and frequency have changed between intervals, separately for each individual islet.

These results seem to suggest that the absence of Epac2A brings about a minor rise in active time during the response to 12 mM glucose and that pharmacological stimulation with forskolin raises the active time through a large increase in oscillation frequency despite a concomitant decrease in oscillation duration. This modulation of [Ca^2+^]_IC_ oscillations is preserved in Epac2A KO mice, which implies that it is most probably largely Epac2A-independent.

#### Functional network parameters

**Figure 4:**
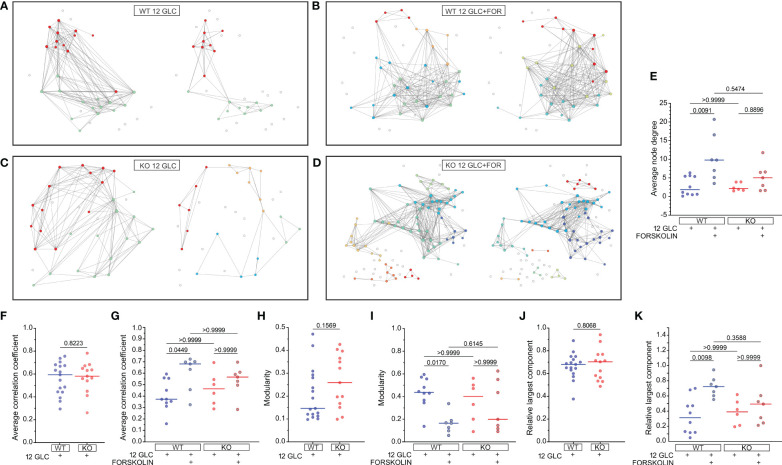
The effect of forskolin on functional beta cell connectivity networks and the role of Epac2A. **(A-D)** Characteristic functional network representations of islets from WT and Epac2A KO mice stimulated either with 12 mM glucose or with 12 mM glucose + 10 µM forskolin. Nodes signify positions of beta cells within an islet, connections stand for functional associations in Ca^2+^ activity, and colors of cells denote different communities. In all four panels the left networks correspond to the first interval in the plateau phase, whilst the right networks represent connectivity patterns in the second interval of sustained activity [i.e., either prolonged glucose stimulation **(A, C)** or stimulation with glucose + forskolin **(B, D)**]; **(E-K)** Synchronization and network metrics for the average pooled data from all islets in the given group: average node degree **(E)**, average correlation coefficient **(F, G)**, modularity **(H, I)**, relative largest component **(J, K)**. In **(F, H, J)** the values in the first part of the plateau phase (glucose only) are shown, whereas in **(E, G, I, K)** the values corresponding to the second part (either glucose only or glucose+forskolin) are presented. Individual dots represent the average values in individual islets, whereas the horizontal lines denote the median value.

To characterize the collective beta cell [Ca^2+^]_IC_ activity, we constructed functional connectivity networks, as described in the Methods section. First, we present characteristic beta cell networks in islets from WT and Epac2A KO mice subjected to 12 mM glucose stimulation only (**Figures 4A, C**) and in islets from both types of mice which were subsequently stimulated with 10 µM forskolin (**Figures 4B, D**). Connectivity maps are presented separately for all four scenarios. After prolonged stimulation with 12 mM glucose only, the networks became much sparser in both WT and KO mice. In contrast, in the second protocol where 10 µM forskolin was added to 12 mM glucose, the networks remained integral, and the number of connections stayed approximately the same. These qualitative observations in the exemplary islets indicate that forskolin rather profoundly affected the collective beta cell behavior by preventing a decline in or even increasing intercellular connectivity, probably to a comparable extent in both types of mice.

To gain a more general and quantitative insight, we show in **Figures 4E-K** different metrics of synchronous beta cell dynamics and network parameters pooled from all islets. We first present the average node degrees, i.e., the average number of functional connections. Since we used an adaptive thresholding approach to set the average degree in the first part in the plateau phase to <*k>*=8 in all islets, we show only the values in the second interval. Results in **Figure 4E** reveal that in both WT and KO mice the average node degree was profoundly lower (median *k*≈2) after prolonged stimulation with 12 mM glucose only. In the stimulation protocol with forskolin, the node degree in the second part of the plateau phase was on average higher in both types of mice when compared to stimulation with glucose only, with median values *k*≈10 and *k*≈5 for the islets from WT and KO mice, respectively. However, the difference between the control and the forskolin protocol was significant only for WT mice.

Next, we show the average correlation coefficients (**Figures 4F, G**), modularity values (**Figures 4H, I**), and relative largest components (**Figures 4J, K**) of beta cell networks. For these metrics, we present first their absolute values in the first part of the plateau phase, separately for the islets from WT and KO mice (**Figures 4F, H, J**). None of these parameters were found to differ between the islets from the WT and KO mice, indicating that under stimulation with 12 mM glucose only, Epac2A does not notably affect either the average synchronicity or the beta cell network’s functional integrity and segregation patterns. We proceeded with examining the differences between the glucose stimulation only protocol and the forskolin protocol by comparing the parameters in the second part of the plateau phase. In the islets which were subsequently stimulated with 10 µM forskolin, the average correlation was higher when compared to prolonged 12 mM glucose stimulation only. Furthermore, the networks were less segregated, and the level of functional integration was greater, as reflected by lower values of modularity (**Figure 4I**) and higher values of the relative largest component (**Figure 4K**), respectively. However, while all the above differences were significant in islets from WT mice, the effect of forskolin was less well-pronounced in islets from Epac2A KO mice and not statistically significant for any of the parameters, suggesting that Epac2A probably importantly contributes to the greater functional integration of cell networks during stimulation by forskolin. To provide a more detailed insight, we additionally present in **Figure S4** how the beta cell synchronization and different network parameters have changed between intervals, separately for each individual islet.

### The effect of forskolin and the role of Epac2A during deactivation of beta cells

**Figure 5:**
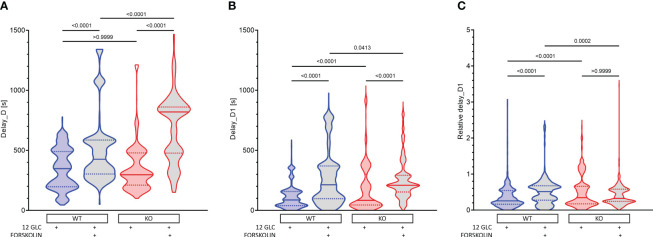
The effect of forskolin and the role of Epac2A during deactivation of beta cells from WT and KO mice. **(A)** The deactivation delays after cessation of stimulation with 12 mM glucose in the presence or absence of 10 µm forskolin. 1^st^ quartile/median/3^rd^ quartile (Q1/M/Q3), in seconds): 197/348/490 (12 mM glucose in WTs), 303/426/586 (12 mM glucose + 10 µM forskolin in WTs), 211/297/479 (12 mM glucose in KOs), and 477/820/861 (12 mM glucose + 10 µM forskolin in KOs); **(B)** The deactivation delays of cells after 1^st^ deactivated cell after cessation of stimulation with 12 mM glucose in the presence or absence of 10 µm forskolin. Q1/M/Q3 (in seconds): 39/87/158 (12 mM glucose in WTs), 97/214/370 (12 mM glucose + 10 µM forskolin in WTs), 45/83/303 (12 mM glucose in KOs), and 153/210/293 (12 mM glucose + 10 µM forskolin in KOs); **(C)** Relative deactivation delays of cells after 1^st^ deactivated cell after cessation of stimulation with 12 mM glucose in the presence or absence of 10 µm forskolin. Q1/M/Q3 (in seconds): 0.16/0.26/0.54 (12 mM glucose in WTs), 0.28/0.51/0.68 (12 mM glucose + 10 µM forskolin in WTs), 0.17/0.34/0.66 (12 mM glucose in KOs), and 0.24/0.32/0.58 (12 mM glucose + 10 µM forskolin in KOs). Data pooled from the following number of cells/islets: 678/13 (12 mM glucose in WTs), 640/10 (12 mM glucose + 10 µM forskolin in WT), 567/10 (12 mM glucose in KOs), 422/7 (12 mM glucose + 10 µM forskolin in KOs). Data were analyzed using one-way ANOVA on ranks (Kruskal-Wallis test) followed by Dunn’s multiple comparisons test, p values are indicated on graphs.

After cessation of stimulation with 12 mM glucose, there was a delay before beta cells became inactive (**Figures 5A**, **S5**). This delay did not differ between WT and KO mice. Additional stimulation by forskolin significantly prolonged the activity of cells (in WTs by 22%, from 348 s to 426 s, **Figure 5A**). This prolongation was not Epac2A-dependent, as it was even significantly longer in KO mice (from 297 to 820s, **Figure 5A**). Thus, at least under conditions of additional stimulation by forskolin, Epac2A seems to restrict the activity of beta cells upon removal of the stimulus. In accordance with our previous studies, comparing deactivation delays between different cells (**Figure 5B**) demonstrated relatively large intercellular differences (43, 64, 81). Stimulation by forskolin enlarged the first-cell-any-cell delays (in WTs from 87 s to 214 s, **Figure 5B**). Although this effect of forskolin did not depend on Epac2A in absolute terms (214 s in WT and 210 s in KO mice, **Figure 5B**), taking into account the inter-islet variability of the median delays and the absolutely longer delays in KOs revealed that forskolin increased the relative heterogeneity only in WTs (from 0.26 to 0.51, **Figure 5C**). On the other hand, normalization with median values of delays also showed that the relative heterogeneity may be slightly higher in KOs after stimulation with glucose only (0.21 vs. 0.34, **Figure 5C**).

Taken together, these results suggest that forskolin increases beta cell activity not only by making the activation more likely and by raising the active time during the plateau phase, but also by prolonging beta cell activity upon removal of the stimulus, extending their activity into an otherwise silent period. This effect of forskolin on deactivation seems to be Epac2A-independent and under normal conditions, Epac2A may even restrict it. Forskolin also made deactivation more heterogenous in absolute terms, but given the huge prolongation of deactivation in KOs, this increase in heterogeneity was relatively larger in WTs. On the other hand, after stimulation with glucose only, the deactivation was in relative terms slightly more heterogenous in KOs.

## Discussion

The principal aims of this study were to describe in detail the possible role of Epac2A and the effects of forskolin on [Ca^2+^]_IC_ oscillations in beta cells during activation, activity, and deactivation, in terms of classical physiological parameters and by using network analyses. First, it should be noted that the differences between Epac2A KO mice and their WT littermates observed in our study are probably not the consequence of altered physiological and biochemical parameters, since previous studies have shown that Epac2A KO mice are generally healthy, with no noticeable physiological abnormalities. Their body weights and food intake are similar as in WT mice, the fasting blood glucose levels and levels of insulin secretion are also similar, and they have normal glucose tolerance and insulin sensitivity (40, 41, 89). On the other hand, the same studies revealed that Epac2A KO mice have larger adipocytes and higher amounts of white adipose tissue, their plasma leptin level is increased, and adiponectin level is decreased already at 7 weeks of age compared with WT mice. Furthermore, these mice are more susceptible to developing obesity when fed a high fat diet. Leptin resistance test performed *in vivo* showed suppressed hypothalamic leptin signaling (40, 89). Since Epac2A is expressed in hypothalamus and heart, but neither in white and brown adipose tissue nor in skeletal muscles, the defect in hypothalamic leptin signaling likely accounts for the disrupted physiological and biochemical parameters observed in Epac2A KO mice when exposed to high metabolic demands (40, 41). Considering the above evidence, we strongly believe that the differences between Epac2A KO mice and their WT littermates observed in our study are at least for the most part due to intrinsic differences in the neurohormonal amplifying pathway of insulin secretion in beta cells due to the lack of Epac2A.

Second, we showed previously that pancreatic beta cells within acute mouse pancreas tissue slices are typically silent in 6 mM glucose and respond to stimulatory glucose concentrations above 7 mM with a transient increase of [Ca^2+^]_IC_ followed by fast [Ca^2+^]_IC_ oscillations (43, 57, 81, 88). In the present work, we demonstrated that beta cells from both Epac2A KO and WT mice responded to the otherwise substimulatory glucose (6 mM) after stimulation by forskolin. This indicates that a rise in cAMP due to adenylyl cyclase activation can affect [Ca^2+^]_IC_, probably in a PKA-dependent and Epac2A-independent manner, as already described before (15, 17, 90). Several studies demonstrated that this could involve an increased cytosolic ATP/ADP ratio, either by accelerated ATP synthesis, decreased ATP degradation, or by redistribution of ATP between different intracellular pools (91), decreased K_ATP_ conductance (92), inhibition of K^+^ currents through voltage-dependent K_V_1.4 channels (17, 90), enhancement of Ca^2+^ signals through VDCC and/or Ca^2+^ mobilization from internal stores via RyR or IP3R (19–22). More studies are required to identify the molecular targets responsible for the above effects of cAMP during activation and quantify their contributions, as well as to determine possible cAMP-independent effects of forskolin. Although forskolin is a potent adenylyl cyclase activator that successfully evoked oscillations of [Ca^2+^]_IC_ in 6 mM glucose, the effect of forskolin in 6 mM glucose seems to be significantly weaker compared to the stimulation with 12 mM glucose, since we observed a much shorter activation delay in 12 mM glucose. This supports previously published data showing progressively shorter activation delay with increasing glucose concentrations (43, 57, 81, 88), a phenomenon most probably resulting from the amount of metabolized glucose needed to activate beta cells or direct cAMP production (93, 94). Furthermore, while in 12 mM glucose most beta cells within an islet responded to stimulation, when stimulated with 6 mM glucose + forskolin, only a minority of beta cells became active (data not shown), demonstrating large differences in their metabolic activity and therefore their sensitivity to glucose also under stimulation by forskolin. Since in our protocol beta cells were exposed to forskolin only for 10 min, we wish to point out the possibility that the majority of beta cells within an islet could eventually be recruited and would respond to prolonged stimulation with forskolin, as seen during exposure to lower stimulatory concentrations of glucose (81, 88). Interestingly, beta cells from Epac2A KO mice responded to both protocols with a shorter activation delay compared to their WT littermates (**Figure 2**). As mentioned before, cAMP can affect [Ca^2+^]_IC_ in a PKA-dependent (15, 17, 90) or an Epac2A-dependent manner (19–22, 33). In Epac2A KO cells, all the available cAMP produced either by forskolin-enhanced adenylyl cyclase activity in substimulatory glucose conditions or endogenously synthesized in beta cells after a high glucose load, will act only through PKA-dependent pathways, increasing for instance the L-type VDCC activity after phosphorylation with PKA (19, 20, 95). Our results point to the possibility that the role of Epac2A may be at least partly inhibitory during activation, that in Epac2A KO mice there is a compensatory upregulation of the stimulatory PKA-dependent mechanisms, or a combination of both. However, further studies are required to confirm and further clarify this mechanism. The activation delays among islets as well as among beta cells within the same islet were very heterogeneous, as observed from the delays between the first-responding cell and the others from the same islet in both protocols (**Figures 2**, **S1**). Most previous studies confirm this, showing at least some degree of heterogeneity between cells during their activation (72, 81, 88, 93, 96–101). Here we showed that in both WT and KO cells, the heterogeneity of delays decreased with faster activation, enabling beta cells a more homogeneous response to a stronger stimulation (i.e., 12 mM glucose vs. 6 mM glucose + 10 µM forskolin). Interestingly, the relative heterogeneity of activation delays was higher in beta cells from Epac2A KO mice in the high glucose regime (**Figure 2C**), suggesting the involvement of Epac2A in the coordination of activation under a high glucose load. The latter could be mediated through Cx36 gap junction coupling in an Epac2A-dependent manner to overcome the extensive intrinsic heterogeneity present in beta cells and to ensure a more coordinated response to glucose (78, 81). This view is also consistent with the above finding that the responses are faster in Epac2A KO mice, since in more weakly coupled syncytia, the intrinsically more sensitive cells can escape the inhibition from less responsive cells (57, 102, 103). Most importantly, our findings regarding network and deactivation properties also argue for the role of Epac2A in intercellular coupling (see below) and are consistent with another recent report (51), but deserve to be explored further in the future.

Next, the plateau phase of response after stimulation with high glucose consists of repetitive fast [Ca^2+^]_IC_ oscillations that reflect bursts of membrane potential depolarizations (46, 97, 104, 105) or Ca^2+^ release from intracellular Ca^2+^ stores (106). Epac2A ablation increased the beta cell active time to a minor extent (**Figure 3**) that is most probably biologically irrelevant, since the Epac2 KO animals display normal insulin and glucose levels, as discussed above. In our hands, the absence of Epac2A did not prevent the forskolin-mediated rise in frequency of oscillations and active time (**Figure 3**). A similar effect was observed for oscillations of membrane potential in GLP-1, forskolin, or glucagon-activated cells under high glucose conditions (92, 107–109). Interestingly, the cells that were previously less active increased their oscillation frequency the most, while the duration of oscillations decreased slightly in the majority of cells (**Figure S2**). This response to forskolin clearly shows that the activation of the neurohormonal amplifying pathway through cAMP involves different molecular mechanisms compared to glucose stimulation, which results in longer oscillation duration at the cost of reduced oscillation frequency or in increased frequency, but without a significant drop in oscillation duration (48, 62, 81, 88). The potentiation of [Ca^2+^]_IC_ oscillations under the influence of forskolin is probably exerted independently of K_ATP_ channels, since GLP-1 is able to increase insulin secretion also in Kir6.2 KO mice that lack functional K_ATP_ channels (110) and probably involves increased Ca^2+^ influx through L-type VDCCs, phosphorylated by PKA (18) as well as PKA- and Epac2A-dependent mobilization of Ca^2+^ from internal stores (19–22, 111). In addition to the possible direct effects of cAMP on [Ca^2+^]_IC_, there is evidence that the amplifying effect of cAMP could be explained by a larger gap junctional conductance between beta cells (70). In beta cell syncytium, electrical coupling through Cx36 plays a crucial role in regulating coordinated glucose induced Ca^2+^ oscillations, thereby determining the dynamics of insulin secretion (75, 112, 113). In the present paper, we demonstrated that under stimulatory glucose levels, electrical coupling results in well-coordinated Ca^2+^ oscillations, but prolonged exposure to glucose causes an Epac2A-independent beta cell desynchronization, lower network integrity and higher segregation (**Figure 4**), as described previously (114). On the other hand, forskolin can prevent this decline in network function, but in an at least partly Epac2A-dependent manner, since in Epac2A KO mice, the improvement in network parameters was only partial. Disrupted cellular communication was described before in metabolically overloaded cells (48, 51, 67, 72, 115), while the GLP-1R agonist Exendin-4 increased Cx36 coupling and improved Ca^2+^ oscillation coordination (51). Several mechanisms have been proposed to explain how cAMP regulates Cx36, either by changing Cx36 gene expression, increasing Cx36 coupling, or by changing distribution of Cx36 on the cell membrane (75, 116, 117). In the retina, cAMP has been shown to regulate gap junction coupling in a PKA-dependent manner (118), with no effect on trafficking or changing the distribution of Cx36 on the plasma membrane (76, 119), while in myocardial cells, PKA is responsible for opening of Cx36 (120). On the other hand, in neurons, regulation of Cx36 is Epac2A-mediated (121). In pancreatic beta cells, both PKA and Epac2A seem to be responsible for Cx36 regulation. PKA was proposed as a channel gating regulator, while Epac2A probably influences Cx36 coupling via slower mechanisms, such as trafficking, assembly, or turnover (51), similarly as in rat myocardial cells (77, 78). Overall, our data suggest that the presence of Epac2A is not critical for basal beta cell functional network integrity upon stimulation with glucose, as no notable differences in beta cell synchronicity and network connectivity were observed when comparing the behavior of control and Epac2A KO islets. However, when the islets were additionally stimulated with forskolin, cells from Epac2A KO mice failed to fully exploit the positive effects of elevated cAMP on beta cell network activity. This might be a consequence of the Epac2A-related deficiency of the connexon trafficking pathways (51, 75, 79, 122). However, additional studies are required to determine the effects of Epac2A deficiency on connexon trafficking in beta cells and to elucidate whether this is the main factor that affects the beta cell network dynamics when cAMP is increased.

Finally, after cessation of stimulation, the oscillatory activity in beta cells gradually stops, and [Ca^2+^]_IC_ returns to baseline. Proper deactivation is especially important to prevent hypoglycemia (102, 123). Like activation, the deactivation phase is also glucose-dependent (81, 88). After decreasing glucose from 12 mM to the substimulatory level (6 mM), beta cells deactivated with 2-3 times greater time lags compared to the activation phase, with no differences between WT and Epac2A KO mice (**Figure 5**). Again, considerable heterogeneity was observed among cells, which is in accordance with previously published studies (43, 64, 81, 88). When beta cells were additionally stimulated by forskolin, the deactivation delay was prolonged significantly, similarly to what can be seen under an extremely high glucose load (81, 88), and this prolongation was especially pronounced in cells lacking Epac2A. Longer and more heterogenous deactivation delays probably indicate a greater degree of activation during the preceding stimulation period, not only through the triggering pathway, but also through the neurohormonal amplifying pathway and probably elevated cAMP levels. It is reasonable to speculate that following strong stimulation, every beta cell will need longer to decrease the concentration of the triggering and amplifying secondary messengers below the stimulatory level. To speculate even further, we believe that slight changes in Cx36 coupling in Epac2A KO mice could at least partly account for the faster and more heterogeneous activation, decreased responsiveness of network parameters to cAMP, and also the longer deactivation delay, since stronger intercellular coupling during deactivation is expected to bring about a stronger hyperpolarizing influence from the already deactivated cells on the still active cells. This hypothesis is a good starting point for future work that should also test the translational relevance of our findings in mouse models of diabetes mellitus and for the human islets, and aim at measuring cAMP dynamics simultaneously with [Ca^2+^]_IC_. With regard to the latter, a concentration of forskolin equal to the concentration used in this study tripled cAMP levels in isolated MIN6 cells (from ~2,5 to 7.5 ng/mg total protein (124)) and rat beta cells (from ~ 0,7 to 2,1 pmol/ml (125)). Experimental quantification of cAMP levels would also enable us to demonstrate explicitly that forskolin indeed elevated cAMP. To our knowledge, there are currently two approaches to measure cAMP levels: lysis of isolated islets (124, 125) and transfection with biosensor-encoding adenoviruses (126). While the first approach is not feasible in the tissue slice (lysis of the tissue slice would include contamination with other cell types, such as acinar and ductal cells), the second approach requires a cultivation period that can critically alter the normal glucose response (127). Based on previously published results, we can thus only assume that cAMP and [Ca^2+^]_IC_ dynamics are tightly correlated (126, 128). We also wish to point out that the most distal part in the stimulus-secretion coupling cascade, i.e., insulin secretion, was not addressed in this study. However, we and others showed previously that cAMP increases the sensitivity of insulin granule fusion or their availability (11, 23–25). The positive Epac2A-independent effect on [Ca^2+^]_IC_ dynamics demonstrated in this study is therefore presumably accompanied also by the effect on the distal steps of the stimulus-secretion coupling cascade. In the future, simultaneous or complementary measurements of secretion dynamics could help us understand the respective contributions of the effects of cAMP on [Ca^2+^]_IC_ and increased sensitivity of the exocytotic apparatus at the level of insulin secretion and determine whether they are additive or synergistic.

## Conclusions

Forskolin was able to weakly activate beta cells exposed to substimulatory glucose and beta cells from Epac2A KO mice responded faster than beta cells from WT littermates to both high glucose and to forskolin added to low glucose. During the plateau phase, forskolin added to high glucose resulted in increased oscillation frequency and relative active time, with well-coordinated activity among beta cells and this response did not critically depend on Epac2A. Prolonged exposure to glucose caused Epac2A-independent beta cell desynchronization, lower network integrity, and higher segregation, while activation of the neurohormonal amplifying pathway prevented this decline in network function in an at least partly Epac2A-dependent manner. In the end, following stimulation with high glucose and forskolin, beta cells deactivated more slowly and the prolongation of activity into the otherwise already silent period was especially well pronounced in beta cells from Epac2A KO mice. Taken together, our results suggest that especially under conditions of stimulated cAMP production, Epac2A may play a role in coordinating beta cell collective activity, with its absence resulting in earlier activation, weaker functional coupling during activity, and later deactivation.

## Data availability statement

The datasets presented in this study can be found in online repositories. The names of the repository/repositories and accession number(s) can be found below: https://doi.org/10.6084/m9.figshare.23723700.v2.

## Ethics statement

The animal study was approved by Administration for Food Safety, Veterinary Sector and Plant Protection of the Republic of Slovenia. The study was conducted in accordance with the local legislation and institutional requirements.

## Author contributions

Conceptualization, MSK, AS, and MSR; methodology, MSK, JD, MG and AS; software, JD and MG; validation, MSK and AS; formal analysis, MSK, JD and MG; investigation, MSK, JD, LK and VP; resources, MSK, JD and MG; data curation, MSK, JD and MG; writing—original draft preparation. MSK, JD, LK, VP, MG and AS; writing—review and editing, AS and MSK; visualization, MSK and MG; supervision, AS and MSR; project administration, MSK, AS and MSR; funding acquisition, AS and MSR. All authors have read and agreed to the published version of the manuscript.

## Supplementary material

Supplementary Video 1 A time series illustrating the response of pancreatic islet cells to 12 mM glucose. Combined movie of average calcium signal (left) and F/F0 (right). F0 was defined as the first 100 frames where no activity was detected. Scale bar = 50 µm. Time stamps were added to the upper right corner.
