# Supplementary material for: The effect of forskolin and the role of Epac2A during activation, activity, and deactivation of beta cell networks
Source: Front Endocrinol (Lausanne). 2023 Aug 28;14:1225486. doi: 10.3389/fendo.2023.1225486 (PMC10494243; doi:10.3389/fendo.2023.1225486)
Supplement: Supplementary file 2 [file Image_1.pdf]

Supplementary Material

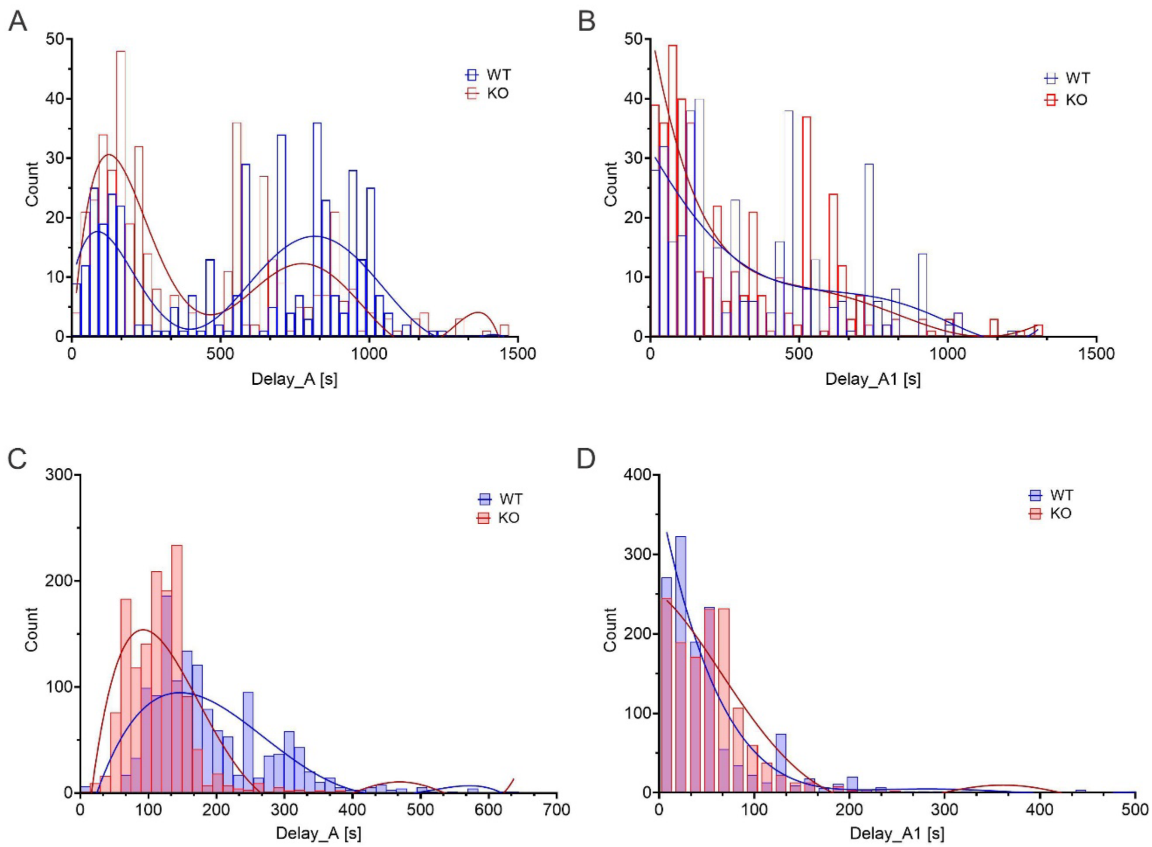

Figure S1: Distribution of activation delays after stimulation with glucose and forskolin in WT and KO mice. (**A-B**) Distribution of activation delays (Delay_A, panel A) and any-cell-first-responder delays (Delay_A1, panel B) after stimulation with 6 mM glucose + 10 μM forskolin; (**C-D**) Distribution of activation delays (panel C) and any-cell-first-responder delays (panel D) after stimulation with 12 mM glucose.

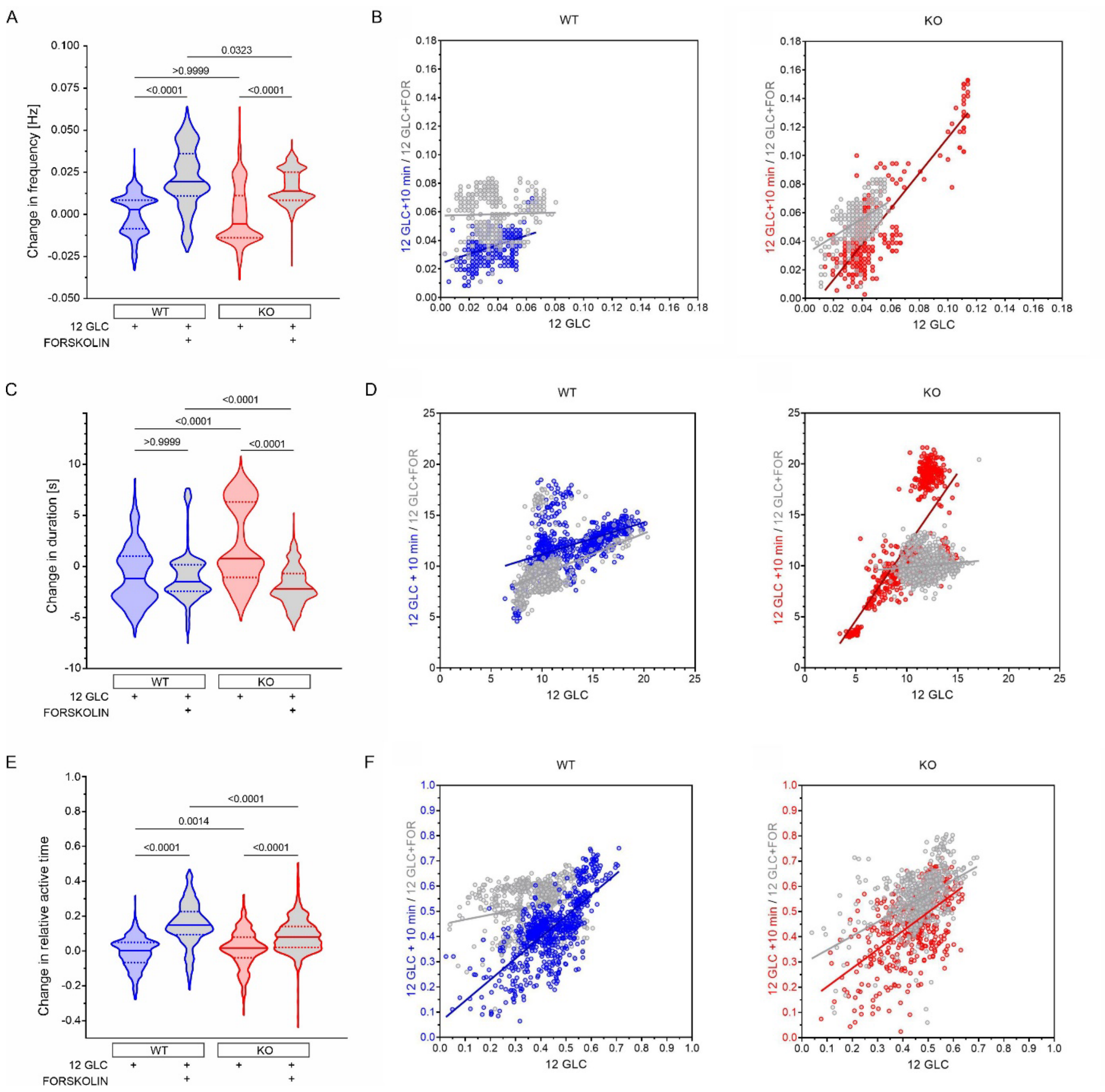

Figure S2: The effect of forskolin on frequency, duration and AT and the role of Epac2A. (**A, C, E**) The differences in (**a**) oscillation frequency, 1st quartile/**median**/3rd quartile (Q1/**M**/Q3) in Hz: -0.008/**0.003**/0.008 (12 mM glucose in WTs), 0.011/**0.020**/0.036 (12 mm glucose + forskolin in WTs), -0.014/**0.006**/0.011 (12 mM glucose in KOs) and 0.08/**0.014**/0.025 (12 mm glucose + forskolin in KOs; (**C**) duration Q1/**M**/Q3 in seconds: -3.1/**-1.2**/1 (12 mM glucose in WTs), -2.4/**-1.5**/0.17 (12 mM glucose + 10 µM forskolin in WTs), -1.1/0.8/0.6.3 (12 mM glucose in KOs) and -3.1/**-2.2**/-0.7 (12 mM glucose + 10 µM forskolin in KOs); and (**E**) relative active time Q1/**M**/Q3: -0.066/**0.003**/0.050 (12 mM glucose in WTs), 0.094/**0.150**/0.225 (12 mM glucose + forskolin in WTs), -0.039/**0.017**/0.078 (12 mM glucose in KOs) and 0.020/**0.080**/0.141 (12 mM glucose + forskolin in KOs) between the first and the second part of the plateau phase, separately for WT and KO mice and for both protocols; (**B, D, F**) Graphs represent the corresponding changes in frequency (**B**), duration (**D**) and relative active time (**F**) in individual cells in both WT and KO mice between the first and the second part of the plateau phase, separately for WT and KO mice and for both protocols. Data pooled from the following number of cells/islets: 687/11 (12 mM glucose in WTs), 656/11 (12 mM glucose + 10 µM forskolin in WTs), 548/7 (12 mM glucose in KOs), and 631/9 (12 mM glucose + 10 µM forskolin in KOs). Data were analyzed using one-way ANOVA on ranks (Kruskal-Wallis test) followed by Dunn's multiple comparisons test, p values are indicated on graphs.

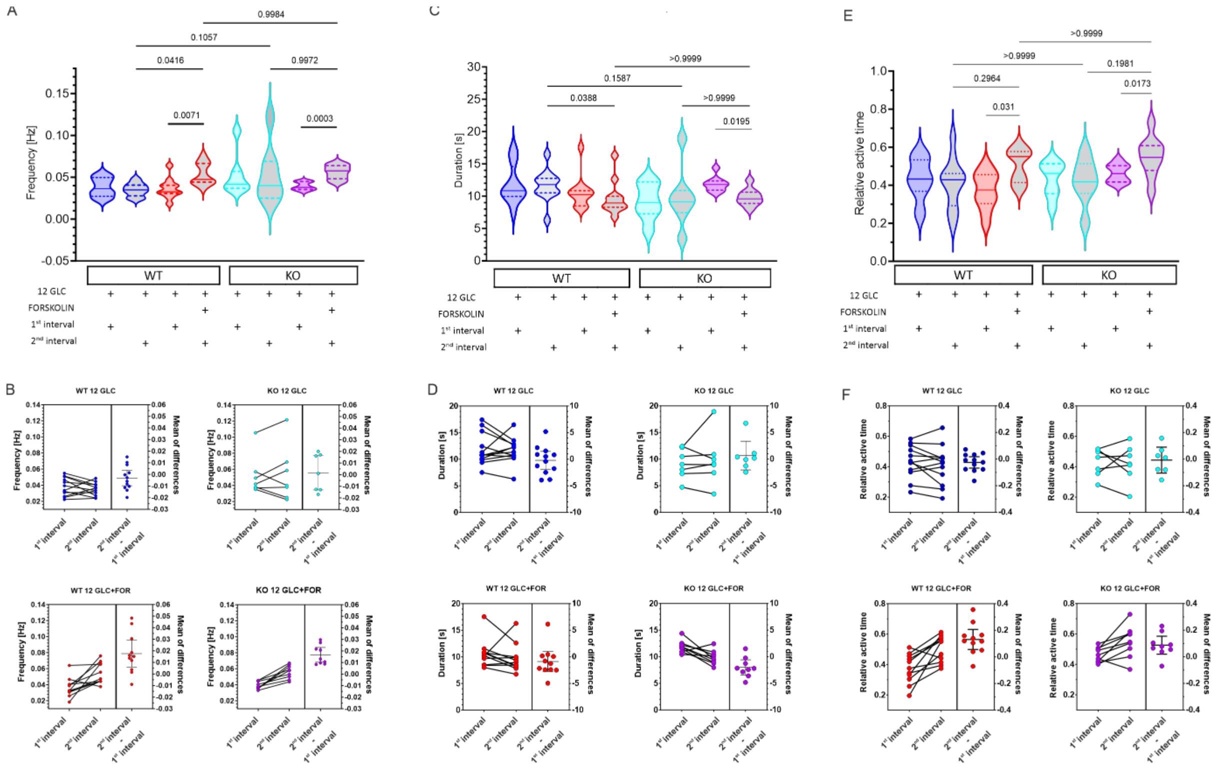

Figure S3: The effect of time and forskolin on frequency, duration and AT in WT and Epac2A KO islets. Panels (**A, C, E**) show oscillation frequency (A), duration (B) and active time (C) during the first and second part of the plateau phase, separately for both types of mice and for both protocols (stimulation with 12 mM glucose only and stimulation with 12 mM glucose + 10 µM forskolin in the second part of the stimulation). The horizontal line denotes the median value while both dashed lines represents the 1st and 3rd quartile. Panels (**B, D, F)** represent the same data as in A, C, D for individual islets during both parts of the plateau phase in different groups. Data pooled from the following number of islets: 12 (12 mM glucose in WTs), 11 (12 mM glucose + 10 µM forskolin in WTs), 7 (12 mM glucose in KOs), and 9 (12 mM glucose + 10 µM forskolin in KOs). Data were analyzed using either paired t-test to compare the two intervals within a single protocol or ordinary one-way ANOVA followed by Šídák's multiple comparisons test for different protocols, p values are indicated on graphs.

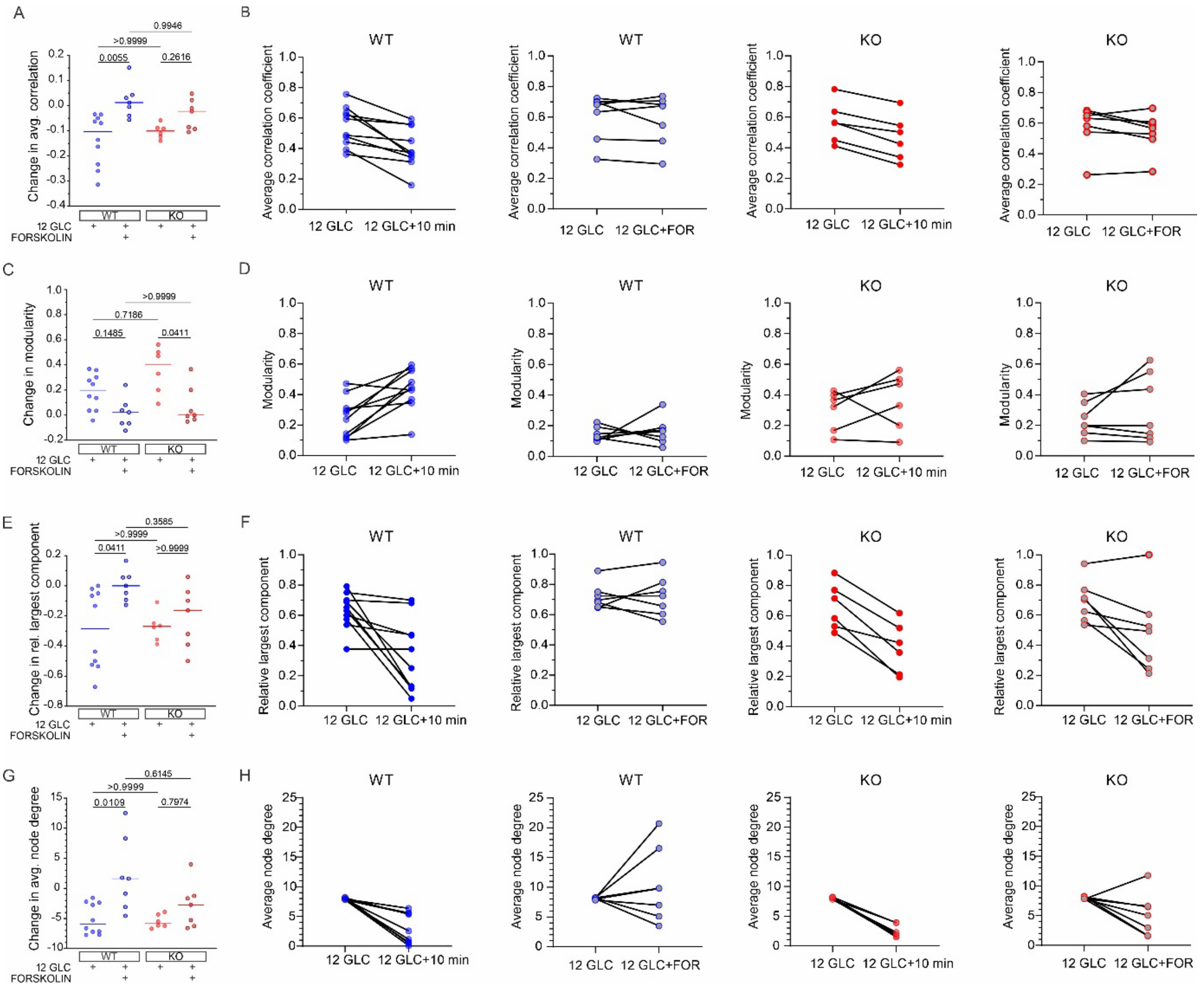

Figure S4: Changes in beta cell synchronicity and functional network parameters. Panels (**A, C, E, G**) denote differences in parameter values between the first and second part of the plateau phase, separately for both types of mice and for both protocols (stimulation with prolonged 12 mM glucose and stimulation with 12 mM glucose + 10 µM forskolin in the second part of the stimulation). Individual dots represent the average values in individual islets, whereas the horizontal line denotes the median value. Panels (**B, D, F, H**) represent the corresponding changes in individual islets in different groups.

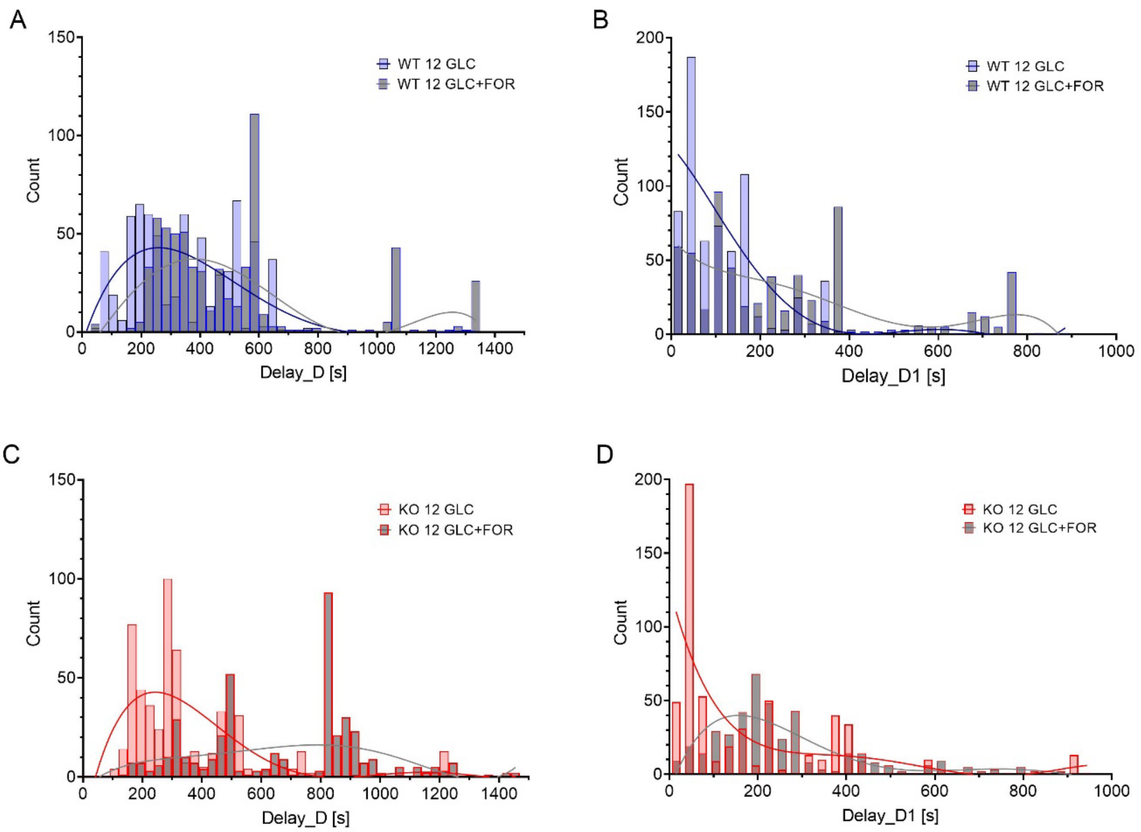

Figure S5: Distribution of deactivation delays after the end of stimulation with glucose and forskolin in WT and KO mice. (**A**) Distribution of deactivation delays after cessation of stimulation with 12 mM glucose (blue) and 12 mM glucose + 10 μM forskolin (grey) in WTs; (**B**) Distribution of the deactivation delays of cells after 1st responded cell after cassation of stimulation with with12 mM glucose (blue) and 12 mM glucose + 10 μM forskolin (grey) in WTs; (**C**) Distribution of deactivation delays after cassation of stimulation with 12 mM glucose (red) and 12 mM glucose + 10 μM forskolin (grey) in KOs; (**D**) Distribution of the deactivation delays of cells after 1st responded cell after cassation of stimulation with with12 mM glucose (red) and 12 mM glucose + 10 μM forskolin (grey) in KOs.
